# Diversity of Bacterial Biofilm Communities on Sprinklers from Dairy Farm Cooling Systems in Israel

**DOI:** 10.1371/journal.pone.0139111

**Published:** 2015-09-25

**Authors:** Nahum Y. Shpigel, Zohar Pasternak, Gilad Factor, Yuval Gottlieb

**Affiliations:** 1 The Koret School of Veterinary Medicine, The Robert H. Smith Faculty of Agriculture, Food and Environment, The Hebrew University of Jerusalem, Rehovot, Israel; 2 Department of Plant Pathology and Microbiology, The Robert H. Smith Faculty of Agriculture, Food and Environment, The Hebrew University of Jerusalem, Rehovot, Israel; 3 Hachaklait, Mutual Society for Veterinary Services, Caesarea Industrial Park, Caesarea, Israel; Loyola University Chicago, UNITED STATES

## Abstract

On dairy farms in hot climates worldwide, cows suffer from heat stress, which is alleviated by the use of water cooling systems. Sprinklers and showerheads are known to support the development of microbial biofilms, which can be a source of infection by pathogenic microorganisms. The aim of this study was to investigate the presence of microbial biofilms in dairy cooling systems, and to analyze their population compositions using culture-independent technique, 16S rRNA gene sequencing. Biofilm samples were collected on eight dairy farms from 40 sprinklers and the microbial constituents were identified by deep sequencing of the 16S rRNA gene. A total of 9,374 operational taxonomic units (OTUs) was obtained from all samples. The mean richness of the samples was 465 ± 268 OTUs which were classified into 26 different phyla; 76% of the reads belonged to only three phyla: Proteobacteria, Actinobacteria and Firmicutes. Although the most prevalent OTUs (*Paracoccus*, *Methyloversatilis*, *Brevundimonas*, *Porphyrobacter*, Gp4, *Mycobacterium*, *Hyphomicrobium*, *Corynebacterium* and *Clostridium*) were shared by all farms, each farm formed a unique microbial pattern. Some known potential human and livestock pathogens were found to be closely related to the OTUs found in this study. This work demonstrates the presence of biofilm in dairy cooling systems which may potentially serve as a live source for microbial pathogens.

## Introduction

**Fig 1:**
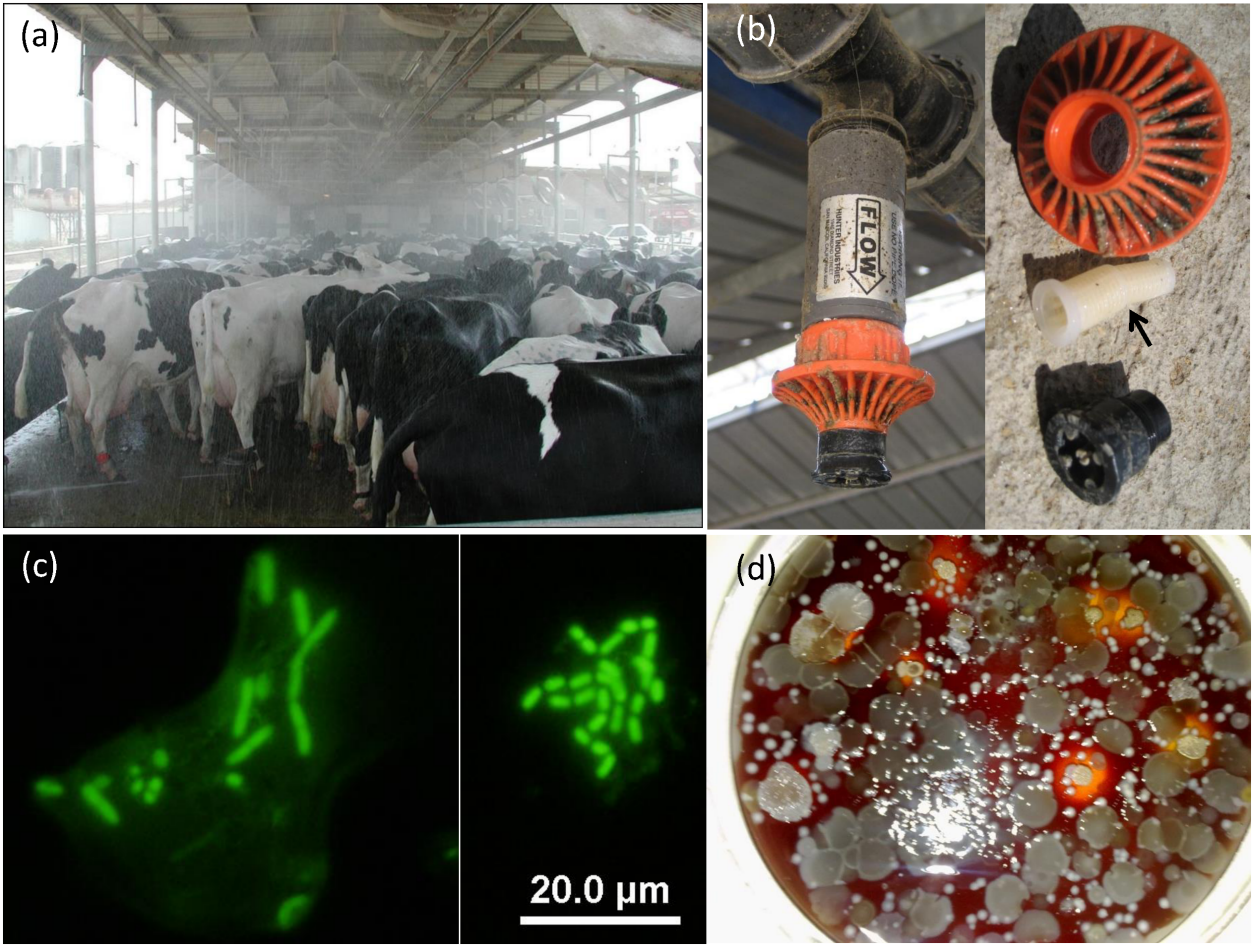
Biofilm in the water sprinklers of dairy farm cooling systems. (a) Typical cooling system in the holding area of a milking parlor. Water sprinklers above the cows are used to soak/wet the cows. (b) Dismantled sprinkler and its filter. (c) Biofilm sampled from sprinkler filter stained with Acridine orange and visualized by epifluorescence microscopy or (d) cultured on blood agar. Scale bar for panel c is 20 μm.

Many dairy farms are located in humid and hot climates where cows suffer from heat stress. This negatively affects feed intake, milk yield, reproduction and welfare [1]. Cooling systems have been successfully developed and implemented in most dairy farms operating in tropical and subtropical areas in an attempt to improve production efficiency and decrease economic losses [2–4]. In highly humid areas like Israel, where annual relative humidity ranges are 41–88% in the northern part of the country and 46–76% in the south (Israel meteorological service: http://www.ims.gov.il/), cooling systems are based on repeated soaking/wetting of the animal's coat followed by forced ventilation to evaporate and extract heat from the body surface [5–7]. Most common in Israel are automated systems of low-pressure sprinklers and fans in the holding area of the milking parlor and in the feeding areas (Fig 1a). These systems are directly supplied with fresh water and the use of recycled, reclaimed or brackish water for cooling is currently prohibited.

Although the economic and welfare benefits of dairy cooling systems are well documented, these systems might also be a source of potential health problems in the herd. Cooling water runoff from the animal's body to the udder and teats might be a source of increased risk for mastitis in dairy cows [8]. The high humidity and slippery concrete floors are expected to affect hoof horn quality, leading to foot infection and lameness [9]. In Israeli dairy herds, the high prevalence of infectious cutaneous diseases such as dermatophilosis and pseudotuberculosis, caused by *Dermatophilus congolensis* and *Corynebacterium pseudotuberculosis* respectively, might be attributed to the extensive adoption of evaporative cooling systems in these herds [10]. Dermatophilosis is known to be more prevalent in tropical and subtropical areas, where heat and humidity contribute to the survival and spread of the organism and probably affect the cutaneous immune response and skin integrity as a barrier to infection [11]. Although it has yet to be demonstrated in cattle, ambient temperature and humidity are known to affect the cutaneous microbiota in humans [12]. The currently used cooling systems might similarly affect cutaneous immunity and microbiota. Furthermore, protective immunity to cutaneous pathogens has been found to be critically dependent on the skin microbiota [13].

Water systems and showerheads are known to support the development of microbial biofilms which can be a source of infection by pathogenic microorganisms, including *Mycobacterium avium* [14, 15]. This bacterium is closely related to *Mycobacterium avium paratuberculosis*, the etiological agent of Johne’s disease in cattle [16, 17]. Hence, we hypothesize that apart from affecting cutaneous integrity and immunity, evaporative cooling systems might be a source of microbial infection for dairy cows. Moreover, sprinkler malfunction is frequently observed in dairy farms, leading to improper wetting of the cows. Biofilms are notorious for affecting devices in water systems and are associated with corrosive damage, clogging and mechanical malfunctions [18]. Clogged filters can break or leak, and if left untreated, can lead to water waste.

The water pipes and sprinklers making up the dairy cooling system have not been investigated for the presence of biofilms. The sprinklers and their filters, much like showerheads, are important interface for interactions between microbes and animals through inhalation of aerosols and immersion of body surface [19]. The aims of this study were to investigate the presence of microbial biofilms in dairy cooling system filters, and analyze the population of these biofilms using culture-independent technique, 16S rRNA gene sequencing [20] with a special focus on the above described pathogens.

## Materials and Methods

### Sampling Locations and Procedures

**Fig 2:**
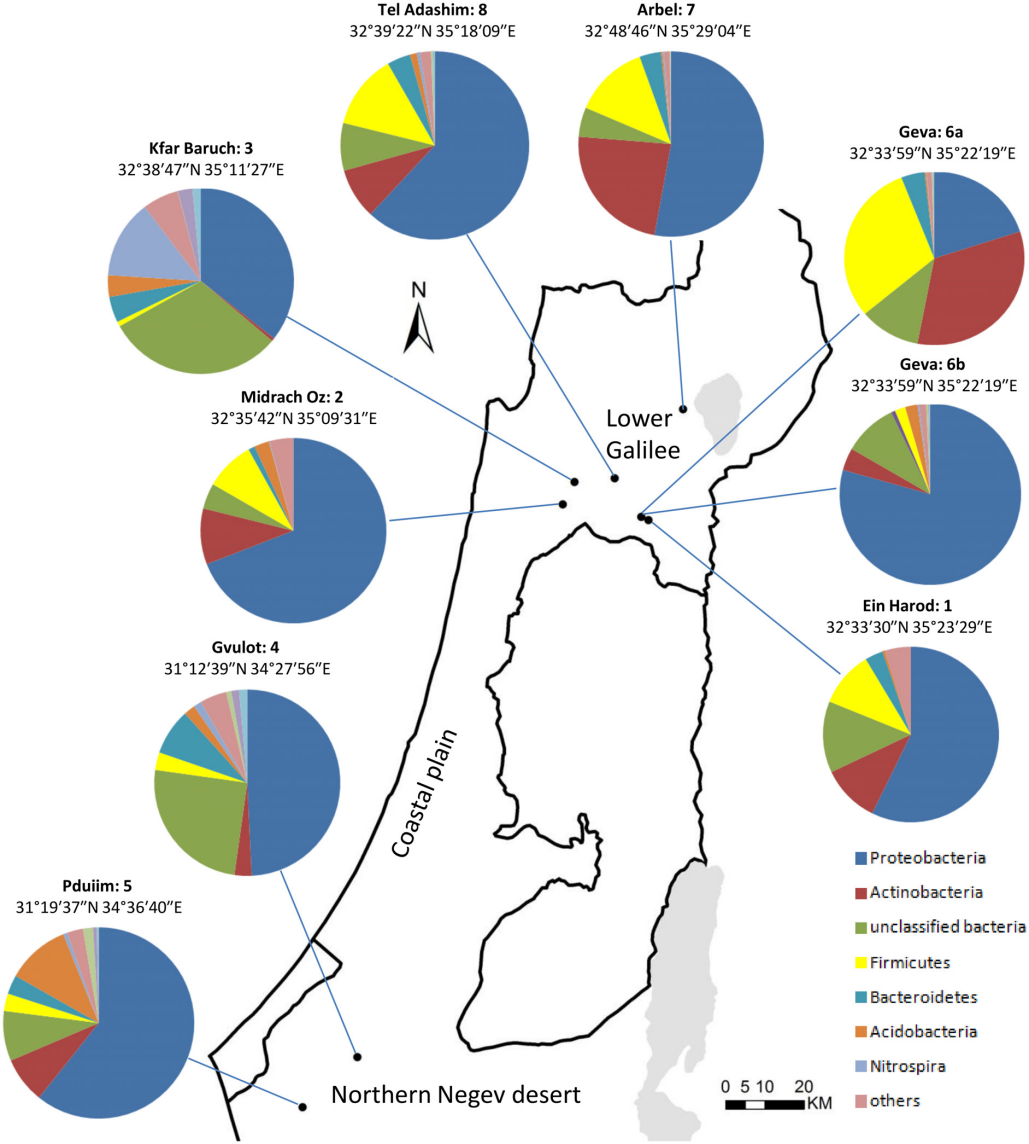
Dairy farms and their microbial abundance. Names and locations (geographic coordinates) of the dairy farms and relative abundance of the most common phyla as revealed by 16S rRNA gene sequencing analysis. The map was constructed using ArcMap 10.0 software (Esri, Redlands, CA).

Biofilm samples were collected on eight dairy farms (Fig 2) from the filters of sprinklers in the holding area of the milking parlor, where cows are cooled before milking and are also brought for additional cooling sessions between milkings (Fig 1a). In one dairy, samples were also collected from sprinklers along the feeding banks (farm 6a and 6b for feeding and holding areas, respectively). These systems are directly supplied with fresh, chlorinated tap water abstracted from a local aquifer (farms 1&6) or lake water supplied by the National Water Carrier (all other farms). A total of 40 sprinkler filters (4 per farm, except farm 6b with 8 samples) were collected in September 2010 while the selected farms experienced outbreaks of pseudotuberculosis and were endemic for dermatophilosis and Johne’s disease (paratuberculosis) (data not shown). The individual sprinkler samples were selected to represent 4 different quadrates within the holding area. To avoid external contamination, biofilms were extracted from the filters in the sprinklers of the cooling system (Fig 1a and 1b) as follows. The filters were aseptically collected into sterile containers and kept on ice until further processing within 1 day. The procedure for biofilm removal and homogenization was performed as previously described [21]. Briefly, 20 mL Ringer’s solution was added to each container which was vortexed for 30 s using REAX top (Heidolph Instruments GmbH, Schwabach, Germany) and was then subjected to sonication for 5 min in an ultrasound bath (Elmasonic S10H, ultrasonic cleaning unit, ultrasonic frequency 37 kHz and effective power 30 W) followed by additional vortexing for 30 s. The sonicated fluid was centrifuged at 10,000*g* for 20 min and the pellet was resuspended in 1 mL PBS and kept frozen for further processing. All samples were collected on private farms under permission granted by farm owners.

### Conventional Microbiological Methods

Culture on blood agar plates (Hylab, Rehovot, Israel) was used to demonstrate the presence of viable and culturable microbes in the disrupted biofilms. Pellet resuspended in PBS was swabbed on agar plates which were incubated for 24 h at 37°C. Suspect isolates for *C*. *pseudotuberculosis* and *D*. *congolensis* were tested with the API-Coryne system (API-bioMerieux) as previously described [22], and as described in [11, 23], respectively.

### Microscopy

Fluorescence microscopy was used to visualize the sprinkler biofilms' microbes. Pellet of disrupted biofilms was swabbed on a glass slide and stained with Acridine orange (5 μg/mL, Sigma, Rehovot, Israel) for 5 min at room temperature. Microbes were visualized and imaged with a Nikon Eclipse E400 epifluorescence microscope and an Olympus DP70 camera.

### Total DNA Extraction and Sequencing

Total DNA was extracted from 1 mL of homogenate using a PowerBiofilm DNA Isolation Kit (MO BIO Laboratories, Inc., Carlsbad, CA) in accordance with the manufacturer’s instructions.

To identify the microbial constituents of the biofilms, 16S rRNA bacterial tag-encoded FLX amplicon pyrosequencing was performed using the Roche 454 sequencing system as previously described [24]. Amplicons originating from the V1–V3 region (27F– 5' GAG TTT GAT CNT GGC TCA G 3' to 519R 5' GTN TTA CNG CGG CKG CTG 3', numbered in relation to the *E*. *coli* 16S rRNA gene), were sequenced.

### 16S rRNA Gene Sequencing Analysis

The 16S rRNA gene sequences (average read length 405 bp) were obtained from a 454 Titanium sequencer (MrDNA, Shallowater, TX). All reads were processed using MOTHUR v1.24 [25]. First, Fasta and quality data were extracted from the raw SFF file. Sequences were grouped according to barcode and primer, allowing one mismatch to the barcode and two mismatches to the primer. Quality control, trimming and de-noising were performed as outlined in the standard MOTHUR 454 protocol (http://www.mothur.org/wiki/454_SOP). All sequences were aligned to the SILVA reference alignment database [25], and filtered so that they all overlap perfectly (with no overhang). To further reduce sequencing errors, sequences were pre-clustered based on the algorithm of Huse et al. [26]. Finally, chimeric reads were removed with MOTHUR’s implementation of the UCHIME method [27], and all chloroplast, mitochondria, and ‘unknown’ (i.e. unclassified at the kingdom level) reads were deleted. This filtering process left ~50% of the sequences, with an average read length of 242 bp.

Pairwise distances were calculated between all DNA reads, and reads were subsequently clustered into operational taxonomic units (OTUs) at the 0.03 level, meaning that sequences that displayed >97% similarity with each other were considered the same OTU. The taxonomic affiliation of each OTU was based on current RDP-II taxonomy [28]. The OTUs were arranged in a data matrix where each row was a single sample and each column a specific OTU; each data point in the matrix represented the abundance of the particular OTU in the particular sample, relativized to the sampling effort (i.e. the number of 454 reads obtained from that sample). Read abundance data were not rarefied, following McMurdie and Holmes [29]. Multivariate analysis was performed in PC-ORD v5.32 (MjM Software, Gleneden Beach, OR) with Sorensen (Bray-Curtis) distances. Ordinations were performed with non-metric multidimensional scaling (NMDS) [30] at 500 iterations, and cluster analyses were performed with flexible beta linkages (β = -0.25). Differences between sample groups were calculated with the multi-response permutation procedure (MRPP) [31], a test based on the assumption that if two groups differ, the average within-group difference is smaller than the average between-group distance. The size of the difference between groups was represented by the A-statistic of the MRPP test, while its significance was identified by the MRPP *P-*value. To determine which OTUs were mainly responsible for differences between groups, we used the method of Dufrene and Legendre [32]. The basis for this procedure is the computation of indicator values (IVs) which are a combination of the frequency of occurrence and abundance of each OTU in each group; the IV ranges between 0 and 100 and is larger if an OTU is more frequent and/or more abundant in a given group compared to another group.

Detailed phylogenetic analysis of individual genera was performed in MEGA5 [33] by compiling all OTU-representative 454 reads from a specific genus together with all long (>1200 bp) type sequences of that genus available at the RDP-II database. Sequences were aligned by MUSCLE [34, 35] and phylogenetic trees were inferred by the maximum likelihood method based on the Tamura–Nei model [36] such that only the tree with the highest log likelihood is shown. Bootstrap values, i.e. the percentage of trees in which the associated taxa clustered together out of 500 repeats, is shown next to the branches. Branches with bootstrap value <50% were collapsed to create polytomy.

## Results

### Demonstration of Microbial Biofilms

Using conventional culture methods and fluorescence microscopy, the presence of live and diverse microbial biofilms in the filters was demonstrated in at least one sample from each farm (Fig 1c and 1d).

### Diversity of Microbial Communities

**Fig 3:**
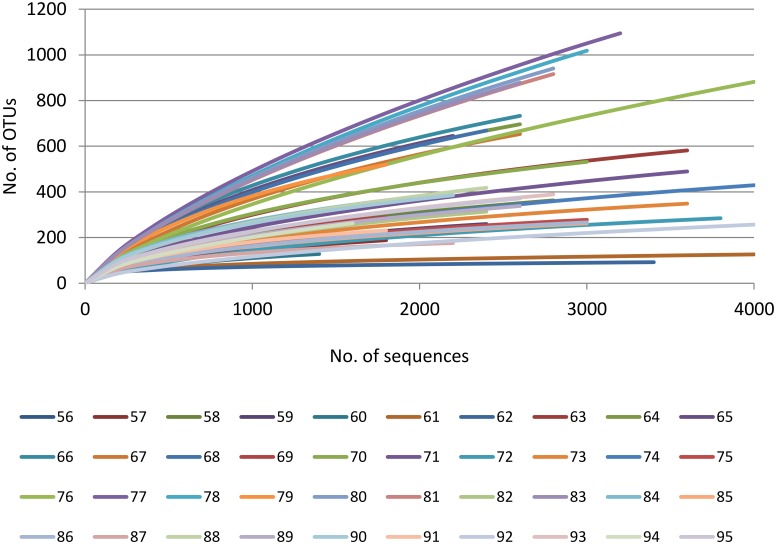
Rarefaction curves of 40 filter samples at a cutoff level of 3%. The rarefaction curve, plotting the number of observed OTUs (sharing ≥97% identity) as a function of the number of sequences, was computed using the RDP Pyrosequencing Pipeline Rarefaction tool.

**Fig 4:**
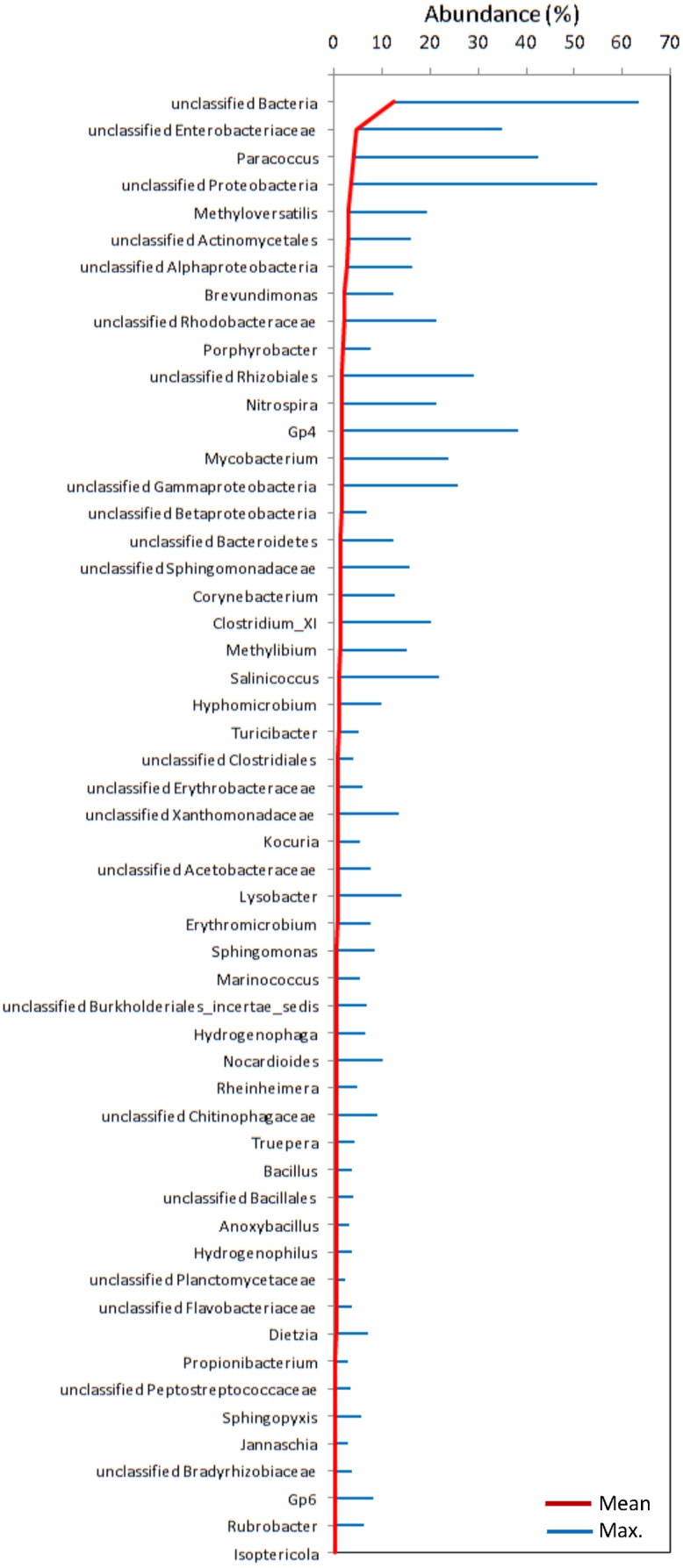
Overall microbial abundance. Mean (red line) and maximum (blue bar) abundance of the most abundant genera in the sprinkler samples.

A total of 113,493 good-quality 454 reads corresponding to 9,374 OTUs at 97% similarity threshold were obtained from all samples. Sequences of the 40 samples were deposited in the MG-RAST database (http://metagenomics.anl.gov/linkin.cgi?project=9157) under accession numbers 4563903.3–4563942.3 (for technical reasons, farm 6b is designated as farm 9 in MG-RAST). Rarefaction curves for each of the 40 samples are presented in Fig 3. For most of the filter samples, the rarefaction curves are close to saturation, confirming the adequacy of our sampling effort and sampling of most of the diversity within the bacterial community. Bacterial taxonomic richness and diversity varied greatly among all samples examined in this study. The mean richness of the samples was 465 OTUs (Range, 93–1101; SD = 268) with a mean evenness of 0.75 (Range, 0.461–0.87; SD = 0.10) and mean Simpson diversity of 0.94 (Range, 0.724–0.99; SD = 0.07). Comparison of OTU richness and Simpson diversity index within and between farms are provided in S1 Table. All OTUs were classified in the domain bacteria, and ~88% were assigned a phylum (i.e. 12% were unclassified bacteria). A total of 26 different phyla were detected in the sprinkler samples, although 76% of the reads belonged to only three phyla: Proteobacteria, Actinobacteria and Firmicutes (Fig 2). Overall, the OTUs belonged to at least 26 different phyla, 49 different classes and 731 genera. The abundance of genera detected in each individual sprinkler sample is illustrated using a heat map (S1 Dataset). The mean and maximum abundance of the most abundant genera are presented in Fig 4.

### Phylogenetic Analysis of Potential Pathogenic Genera

From the OTUs obtained in each sample, we further focused our analyses on the OTUs of the three potentially pathogenic genera *Mycobacterium*, *Corynebacterium* and *Dermatophilus*. OTUs of the genus *Dermatophilus* were not found in the samples. The relationships between OTUs and species of *Corynebacterium* and *Mycobacterium* are shown in S1 and S2 Figs, respectively. None of the OTUs were closely related to *Mycobacterium avium paratuberculosis* or *C*. *pseudotuberculosis*. Furthermore, the presence of the two pathogens *C*. *pseudotuberculosis* and *Dermatophilus congolensis* was not detected by conventional culture (data not shown). Other known environmental mycobacteria, albeit rare, opportunistic pathogens, such as *Mycobacterium phocaicum*, *M*. *austroafricanum* and *C*. *tuberculostearicum*, were found to be closely related to the OTUs found in this study.

### Farm Inventory Diversity

To facilitate the comparison, the sequencing results were also grouped according to farm of origin. Information regarding the mean abundance of genera in each group of samples (farms 1–8) is illustrated with the use of a heat map (S1 Dataset). The most prevalent OTUs that were shared by all sampled farms (core OTUs; farm prevalence = 100%) were identified as member of *Paracoccus* (mean abundance = 4.5%), *Methyloversatilis* (3.3%), *Brevundimonas* (2.5%), *Porphyrobacter* (2.0%), Gp4 (1.9%), *Mycobacterium* (1.9%), *Hyphomicrobium* (1.3%), *Corynebacterium* (1.2%) and *Clostridium*_XI (1.0%).

**Fig 5:**
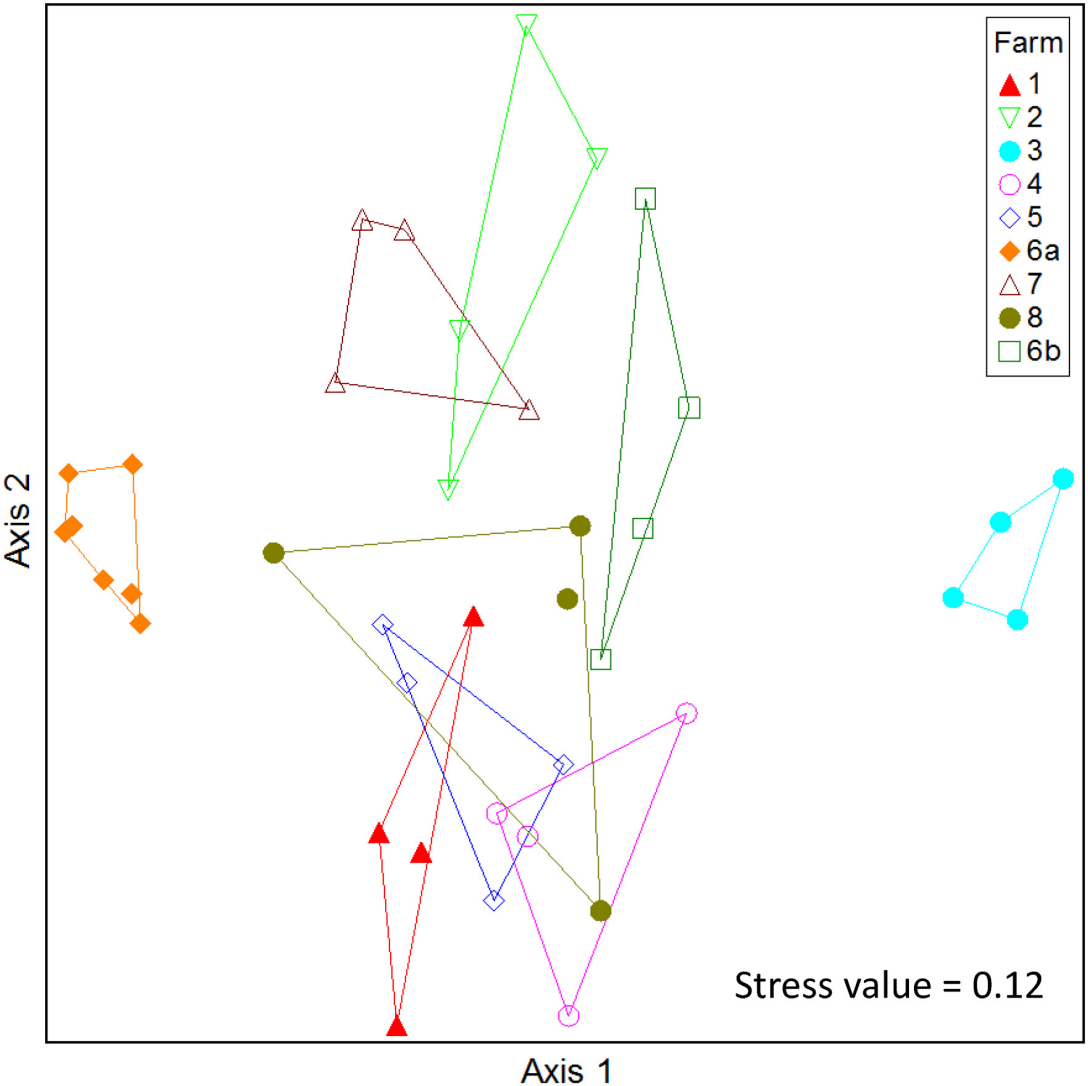
Comparison of bacterial communities from sprinkler samples. NMDS ordination plot comparing sprinkler bacterial communities from different dairy farms (1–8); farm 6a and farm 6b denote samples obtained from the feeding bank and holding area, respectively, on the same farm. Each data point in the NMDS plot represents the bacterial community identified from a single sprinkler sample. Convex hulls are drawn for each farm representing the smallest convex polygon containing all points. Comparison using MRPP revealed a significant difference between the farm samples (*P* = 0.005).

NMDS and MRPP analyses were used to compare the bacterial communities among farms. In addition, bacterial phyla, classes and genera that best distinguish the farms were identified using the Dufrene and Legendre indicator analysis [27]. The NMDS and MRPP analyses showed that the microbial community in each farm differs significantly from that in all other farms (MRPP tests, A = 0.183 ± 0.091, *P* = 0.005 ± 0.003, Fig 5 and S2 Table). Interestingly, the microbial communities in the samples taken at two different sites within the same farm—feeding banks (farm 6a) and holding area (farm 6b)—were also significantly different (A = 0.208, *P* = 0.0047, Fig 5). Identified genera with IV ≥ 90 (*P* < 0.002) for each farm (except farm 2) are presented in S3 Table.

## Discussion

Here we show for the first time the presence of multispecies bacterial biofilms in 40 samples from sprinklers in eight dairy cooling systems. This comprehensive survey, although performed in one time point only, revealed overall rich and diverse bacterial communities. Our results show high mean richness and diversity in comparison to other water-related systems, although this difference may be due to different methodologies used to analyze bacterial communities. Mean richness of the samples was 465, which is higher compared with the estimated richness in other water-related systems using different sequencing protocols, such as 55 in cold drinking water [37], 341 in water meters [38], and 178 in clear well water [39]. The Simpson diversity, 0.94, was also higher in the cooling system than in clear well water (0.27–0.81) [39], or water-distribution systems (less than 0.1) [40].

The core OTUs found in this study belonged to genera that were also found in biofilms recovered from various water systems, e.g. *Paracoccus* [41], *Methyloversatilis* [42], *Brevundimonas* [43], *Porphyrobacter* [44], Gp4 [42], *Hyphomicrobium* [45], *Mycobacterium* [19], *Corynebacterium* [41] and *Clostridium* [46].

Some of these core OTUs may be potential pathogens, as previously shown for mycobacteria in showerheads [19] and drinking water-distribution systems [47], and we therefore further analyzed the OTUs of *Corynebacterium* and *Mycobacterium* for phylogenetic relationships with known species in their genera. Using this method, we showed several OTUs which are related to known potential human pathogens of the non-tuberculosis *Mycobacteria* group such as *M*. *bolletii*, *M*. *parafortuitum*, *M*. *austroafricanum*, *M*. *novocastrense* and *M*. *phocaicum* [48]. Further analysis is needed to confirm the specific species identified in our study.

Phylogenetic analysis of *Corynebacterium* OTUs did not show relationship with known pathogens including *C*. *pseudotuberculosis*. Moreover, *Dermatophilus congolensis* could not be detected in any of the tested filters. Although cutaneous dermatophilosis and pseudotuberculosis are highly prevalent on Israeli dairy farms and have been associated with hot weather and moist conditions, we could not conclude that the bacterial biofilms in the cooling systems serve as a source for cows’ infection with these pathogens. Moreover, granted that none of the above pathogens were identified, the microbial communities found in this study might not represent a random sample of the Israeli dairy farms. Nevertheless, the microbial community in each filter clustered with other filters on the same farm (i.e. not significantly different), and each farm was found to be significantly different from the others. This high inter-farm diversity may not represent the overall diversity, but support a general trend of specific farm holding area community. Other studies have shown that different samples consist of different bacterial communities, and that community structure is dependent on the water source, or on the biofilm age [40, 42]. Those studies, however, focused on only three or four samples. The differences among farms obtained in the current study may stem from the different sources of the water reaching each farm; however, at least two of the farms had the same water source (G. Bernstein, Israel Water Authority, personal communication), and in farm 6, samples taken from two different locations differed significantly in their bacterial communities (P < 0.001). This may result from the filter biofilm's age, or from the specific location of the filters on the farm which might be affected by specific environmental conditions, such as exposure to sun and radiation or wind, or external contamination of the sprinkler. Differences in the microbial community structure of water have been shown to occur with changes in season, temperature and chemical composition [37, 47]. For each farm, except farm 2, strict IV analysis revealed bacterial genera that are found mostly in water-treatment facilities or in natural waterbeds or soil [44, 49, 50]. Further studies with larger sample size and more sampling time points are needed in order to identify bacterial community terns and dynamics and the effects of environmental parameters such as season, water sources and cooling system materials and mode of operation.

This work demonstrates that cooling systems can potentially serve as a source of live microbial biofilm when using high-quality chlorinated drinking water. Despite the surging prices and scarcity of water in our region, we should approach the use of brackish, recycled or low-microbial-level water in dairy cooling systems with great caution, while taking into account the accidental use of contaminated water due to backflow from other malfunctioning water systems.

## Supporting Information

S1 Dataset Heat map of all genera in each sprinkler sample and on each dairy farm. (XLSX)

S1 Table OTU richness and Simpson diversity index of farms. (PDF)

S2 Table Pairwise MRPP values denoting differences between bacterial communities at different farms. (PDF)

S3 Table Indicator values (IV) ≥ 90 of identified genera in each farm. (PDF)

S1 Fig Phylogenetic analysis. Phylogenetic dendrogram derived from 16S rRNA gene sequences of all species of the genera *Corynebacterium* described to date, showing the position of the OTUs among phylogenetic neighbors. Bootstrap values ≥50% based on 500 replications are shown at the branch nodes. OTUs are designated by OTU code number∣total number of reads∣sample number∣farm number.
(TIF)

S2 Fig Phylogenetic analysis. Phylogenetic dendrogram derived from 16S rRNA gene sequences of all species of the genera *Mycobacterium* described to date, showing the position of the OTUs among phylogenetic neighbors. Bootstrap values ≥50% based on 500 replications are shown at the branch nodes. OTUs are designated by OTU code number∣total number of reads∣sample number∣farm number.
(TIF)
