# Supplementary material for: Diversity of Bacterial Biofilm Communities on Sprinklers from Dairy Farm Cooling Systems in Israel
Source: PLoS One. 2015 Sep 25;10(9):e0139111. doi: 10.1371/journal.pone.0139111 (PMC4634551; doi:10.1371/journal.pone.0139111)
Supplement: S1 Table — (PDF) [file pone.0139111.s002.pdf]

S1 Table. OTU richness and Simpson diversity index of farms.

	OTU richness		Simpson diversity	
Farm	Mean	SD	Mean	SD
1	190	46	0.95	0.05
2	153	146	0.88	0.11
3	482	30	0.98	0.02
4	350	208	0.97	0.02
5	269	40	0.88	0.11
6a	466	117	0.97	0.02
7	195	57	0.91	0.05
8	254	46	0.97	0.01
6b	239	44	0.88	0.12
