# Supplementary material for: Diversity of Bacterial Biofilm Communities on Sprinklers from Dairy Farm Cooling Systems in Israel
Source: PLoS One. 2015 Sep 25;10(9):e0139111. doi: 10.1371/journal.pone.0139111 (PMC4634551; doi:10.1371/journal.pone.0139111)
Supplement: S2 Table — (PDF) [file pone.0139111.s003.pdf]

S2 Table. Pairwise MRPP values denoting differences between bacterial communities at different farms.

Farms being compared			A	*P*
3	vs.	6a	0.313546	0.000638
1	vs.	6a	0.23385	0.001014
4	vs.	6a	0.216955	0.001612
5	vs.	6a	0.181735	0.001894
6a	vs.	7	0.159959	0.002601
2	vs.	6a	0.170179	0.002939
6a	vs.	8	0.156614	0.004714
6a	vs.	6b	0.207907	0.004786
8	vs.	6b	0.091374	0.005202
1	vs.	2	0.111171	0.005397
3	vs.	6b	0.288914	0.00541
2	vs.	7	0.051655	0.005442
1	vs.	7	0.121353	0.005447
4	vs.	6b	0.159261	0.005512
2	vs.	6b	0.097277	0.005572
1	vs.	3	0.325931	0.005604
3	vs.	5	0.263165	0.005617
3	vs.	7	0.234036	0.005632
2	vs.	3	0.250495	0.005657
1	vs.	6b	0.192798	0.005672
5	vs.	6b	0.119197	0.005739
1	vs.	5	0.14685	0.005775
3	vs.	8	0.246874	0.005804
2	vs.	4	0.131525	0.005859
1	vs.	4	0.151176	0.005867
7	vs.	6b	0.115868	0.005922
4	vs.	5	0.110138	0.005961
4	vs.	7	0.131344	0.006163
3	vs.	4	0.267706	0.006577
5	vs.	7	0.0918	0.006866
4	vs.	8	0.103934	0.007612
5	vs.	8	0.079756	0.007783
1	vs.	8	0.123036	0.007972
2	vs.	8	0.076638	0.009251
2	vs.	5	0.096025	0.010942
7	vs.	8	0.075979	0.013133
