# Supplementary material for: Diversity of Bacterial Biofilm Communities on Sprinklers from Dairy Farm Cooling Systems in Israel
Source: PLoS One. 2015 Sep 25;10(9):e0139111. doi: 10.1371/journal.pone.0139111 (PMC4634551; doi:10.1371/journal.pone.0139111)
Supplement: S3 Table — (PDF) [file pone.0139111.s004.pdf]

S3 Table. Indicator values (IV) ≥ 90 of identified genera in each farm.

Farm	Genera	IV	Mean	S.D.	p
1	Rubrobacter	95.3	22.4	11.49	0.0002
3	Gemmatimonas	100	16.3	10.19	0.0004
3	Gp6	95.6	19.6	10.57	0.0004
3	Hydrogenophaga	94.3	18.7	10.14	0.0004
3	Leptonema	100	16.6	9.9	0.0004
3	Opitutus	96.5	17.4	9.94	0.0004
3	Pasteuria	100	17.9	9.86	0.0004
3	Thauera	100	16.9	9.96	0.0004
3	Thermomonas	91	17.6	9.7	0.0004
4	Lysobacter	92.4	24.6	12.35	0.0006
4	Rubellimicrobium	100	20	10.49	0.0004
4	Sphingomonas	100	21.5	10.85	0.0004
5	Gp4	95.5	27	12.4	0.0014
5	Lysobacter	91	16.4	9.29	0.0006
5	Methylobacterium	100	19.2	10.05	0.0006
6	Brevibacterium	100	17.6	8.96	0.0002
6	Caryophanon	95.2	17.6	8.43	0.0002
6	Corynebacterium	100	16.6	8.11	0.0002
6	Demequina	100	18.4	9.71	0.0002
6	Dietzia	100	16.9	8.34	0.0002
6	Iamia	92.8	17.9	8.71	0.0002
6	Isoptericola	93.4	21.1	8.17	0.0002
6	Janibacter	91.4	17.8	7.67	0.0002
6	Jeotgalicoccus	100	18.5	9.6	0.0002
6	Joostella	100	16.8	8.24	0.0002
6	Luteimonas	94.8	18.1	8.35	0.0002
6	Nesterenkonia	100	17	8.38	0.0002
6	Nitriliruptor	94.5	18.3	8.06	0.0002
6	Ornithinimicrobium	100	16.9	8.28	0.0002
6	Planomicrobium	100	18	9.3	0.0002
6	Pseudomonas	100	17.9	9.3	0.0002
6	Salinicoccus	100	16.6	8.04	0.0002
6	Yaniella	97.4	19.8	10.3	0.0002
7	Mycobacterium	95.6	25.8	12.8	0.0002
8	Rheinheimera	96.3	25.3	12.53	0.0004
9	Paracoccus	95.5	22	10.98	0.0002
